# A SINE-Derived Element Constitutes a Unique Modular Enhancer for Mammalian Diencephalic *Fgf8*

**DOI:** 10.1371/journal.pone.0043785

**Published:** 2012-08-24

**Authors:** Akiko Nakanishi, Naoki Kobayashi, Asuka Suzuki-Hirano, Hidenori Nishihara, Takeshi Sasaki, Mika Hirakawa, Kenta Sumiyama, Tomomi Shimogori, Norihiro Okada

**Affiliations:** 1 Graduate School of Bioscience and Biotechnology, Tokyo Institute of Technology, Midori-ku, Yokohama, Kanagawa, Japan; 2 RIKEN Brain Science Institute, Hirosawa, Wako City, Saitama, Japan; 3 Bioinformatics Center, Institute for Chemical Research, Kyoto University, Gokasho, Uji, Kyoto, Japan; 4 National Institute of Genetics, Mishima, Shizuoka, Japan; Louisiana State University, United States of America

## Abstract

Transposable elements, including short interspersed repetitive elements (SINEs), comprise nearly half the mammalian genome. Moreover, they are a major source of conserved non-coding elements (CNEs), which play important functional roles in regulating development-related genes, such as enhancing and silencing, serving for the diversification of morphological and physiological features among species. We previously reported a novel SINE family, AmnSINE1, as part of mammalian-specific CNEs. One AmnSINE1 locus, named AS071, showed an enhancer property in the developing mouse diencephalon. Indeed, AS071 appears to recapitulate the expression of diencephalic fibroblast growth factor 8 (*Fgf8*). Here we established three independent lines of AS071-transgenic mice and performed detailed expression profiling of AS071-enhanced *lacZ* in comparison with that of *Fgf8* across embryonic stages. We demonstrate that AS071 is a distal enhancer that directs *Fgf8* expression in the developing diencephalon. Furthermore, enhancer assays with constructs encoding partially deleted AS071 sequence revealed a unique modular organization in which AS071 contains at least three functionally distinct sub-elements that cooperatively direct the enhancer activity in three diencephalic domains, namely the dorsal midline and the lateral wall of the diencephalon, and the ventral midline of the hypothalamus. Interestingly, the AmnSINE1-derived sub-element was found to specify the enhancer activity to the ventral midline of the hypothalamus. To our knowledge, this is the first discovery of an enhancer element that could be separated into respective sub-elements that determine regional specificity and/or the core enhancing activity. These results potentiate our understanding of the evolution of retroposon-derived *cis*-regulatory elements as well as the basis for future studies of the molecular mechanism underlying the determination of domain-specificity of an enhancer.

## Introduction

Retroposons, including short interspersed repetitive elements (SINEs) and long interspersed repetitive elements (LINEs), can proliferate in a genome via reverse-transcription of an RNA intermediate and subsequent integration into random sites in the genome. This ‘copy and paste’ mechanism of amplification is called retrotransposition. Retroposons and their remnants constitute a substantial proportion of vertebrate genomes, e.g., >40% of the human genome [1]. Since the discovery of transposable elements in 1950 [2], they have generally been regarded as ‘junk DNA’. Nevertheless, because retrotransposition can extensively alter genome structure [3], it has been proposed that retroposons might have had a great impact on host genomes and evolution [4].

It is widely accepted that the number and type of coding genes do not vary greatly among vertebrate species from fish to mammals and that protein-coding regions constitute only a small faction of each genome (1.2% [1]). These facts underscore the potentially important role of non-coding regions in gene regulation. This concept has become firmly established with the discovery of a large number of non-coding elements that are highly conserved across species [5]–[8]. These conserved non-coding elements (CNEs) are assumed to be involved in generating different morphological and physiological features among species. They are often conserved only in a certain group of animals (clade-specific conservation; [9]–[11]), implying that they may have important genomic functions responsible for generating clade-specific phenotypes. Several studies have identified many CNEs as *cis*-regulatory elements, mainly as enhancers [9], [12], [13]. In most cases, however, their evolutionary origins and functional role(s) leading to morphological differences among species have not been studied in detail.

One of the most striking discoveries in recent comparative genomics was that transposable elements, including retroposons, are the major source of CNEs [14]–[16]. Indeed, at least 16% of eutherian-specific CNEs were derived from ancient transposable elements [17]. This phenomenon, i.e., that transposable elements acquired new functions after their insertion, is called exaptation [18], and these exapted transposable elements might have acted as a driving force in mammalian evolution by providing novel patterns of gene expression. In this regard, it has been demonstrated that several CNEs, acting as enhancers, were derived from retroposons such as the LF-SINEs, AmnSINE1 and MIRs [19] [20] [21] [22] In most cases, an exapted SINE constitutes only a part of the enhancer element. What function the SINE-derived sequence has and how it together with its flanking sequences acquired an enhancer function during evolution have not been investigated.

We previously characterized a SINE family, AmnSINE1, that is distributed in the genomes of amniotes, including mammals, birds, and reptiles [20]. AmnSINE1s originated in a common ancestor of Amniota, and we found that more than 100 copies of AmnSINE1s make up a part of mammalian-specific CNEs [20] [23]. This finding led us to propose that these AmnSINE1s acquired novel genomic functions in the mammalian common ancestor and are responsible for mammalian-specific characteristics. To examine this hypothesis, we used transgenic mice to test the ability of many AmnSINE1 loci to act as enhancers. In two studies, we identified two AmnSINE1 loci that function as enhancers in the developing mammalian brain [24] [25]. Among them, AS071 showed enhancer activity in the hypothalamus as well as in the dorsal midline and the lateral wall of the diencephalon in the transgenic mouse embryos at embryonic day 10.5 (E10.5) and E11.5. Because this enhancer activity closely resembled the expression pattern of fibroblast growth factor 8 (*Fgf8*), located 178 kb from AS071, we speculated that AS071 might serve as a mammalian-specific distal enhancer for diencephalic *Fgf8* expression [24].

Fgf8, the most studied member of FGF family, is expressed in various organs such as heart, limbs, and head regions (including brain) from early developmental stages, and it acts as a crucial diffusible morphogen in organogenesis during vertebrate development [26]–[34]. Several domains of *Fgf8* expression in the developing brain are known as important organizing centers. For example, *Fgf8* expression in the anterior neural ridge and midbrain-hindbrain boundary is essential for cortical area patterning and midbrain-hindbrain development, respectively [29], [30], [35]–[37]. In contrast to the well-described functions at these two prominent domains, the function of *Fgf8* expression in the diencephalon, including the hypothalamus, has been less studied.

We previously found that *Fgf8* expression in the diencephalon is relatively strong in mouse compared to chick [24], [38]. These data, together with the finding that AS071 constitutes a mammalian-specific CNE, led us to hypothesize that AS071 might contribute to the observed mammalian-specific increase in diencephalic *Fgf8* expression and to mammalian-specific brain formation. Verification of this hypothesis would require further analysis of the mechanism by which AS071 enhances *Fgf8* expression. Also, we aimed to demonstrate the significance of retroposons in the evolution of transcriptional regulatory elements by elucidating the contribution of a SINE to enhancer activity of AS071. We discovered a unique aspect of AS071 in gene regulation, in which the SINE-derived element contributes to conferring enhancer activity to the hypothalamus, providing a new clue to understand the importance of retroposons in the acquisition of *cis*-regulatory elements during evolution. We here propose the possible impact of enhanced *Fgf8* expression in the mammalian diencephalon and its contribution to mammalian-specific traits.

## Results

### Enhancer Activity of AS071 Recapitulates *Fgf8* Expression in the Developing Diencephalon

**Figure 1:**
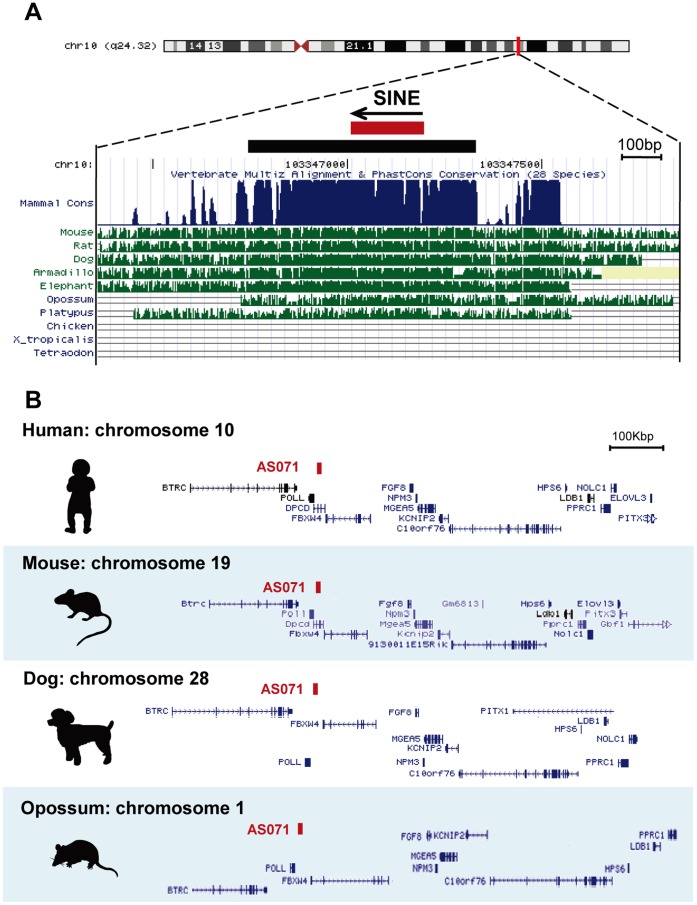
Conservation of the sequence and surrounding gene synteny of AS071. (A) The human AS071 (chr10∶103,356,749–103,357,337, GRCh37/hg19) in the UCSC Genome Browser (http://genome.ucsc.edu/). The red bar corresponds to the AmnSINE1-derived region (homologous to the AmnSINE1 consensus sequence, 204 bp), and the black bar shows the region conserved among mammals (590 bp). Sequence alignment of the region is shown in Figure S1. (B) The conserved gene synteny of the 1-Mb region around the locus among human (chr10∶103,000,000–104,000,000, GRCh37/hg19), mouse (chr19∶45,310,000–46,310,000, NCBI37/mm9), dog (chr28∶16,900,000–17,900,000, Broad/canFam2), and opossum (chr1∶110,001,000–111,001,000, Broad/monDom5). Note the inversion in this region in opossum. Gene annotations are based on the human assembly in the UCSC Genome Browser.

AS071 (589 bp) on human chromosome 10 (Chr10∶103,356,749–103,357,337, GRCh37/hg19) contains a 204 bp region derived from AmnSINE1 (Figure 1A). The sequence of AS071 is highly conserved among all mammals including platypus but not found in other animals, such as birds, amphibians, and fishes (Figure 1A, Figure S1), suggesting an evolutionarily conserved function. Notably, the synteny of genes surrounding AS071 is also highly conserved (Figure 1B). We previously performed enhancer assays using transient transgenic mice into which we introduced 1.5 kb of mouse sequence containing AS071 connected to the *lacZ* reporter gene [24]. We demonstrated that *lacZ* expression driven by AS071 is similar to that of *Fgf8* in the developing diencephalon.

**Figure 2:**
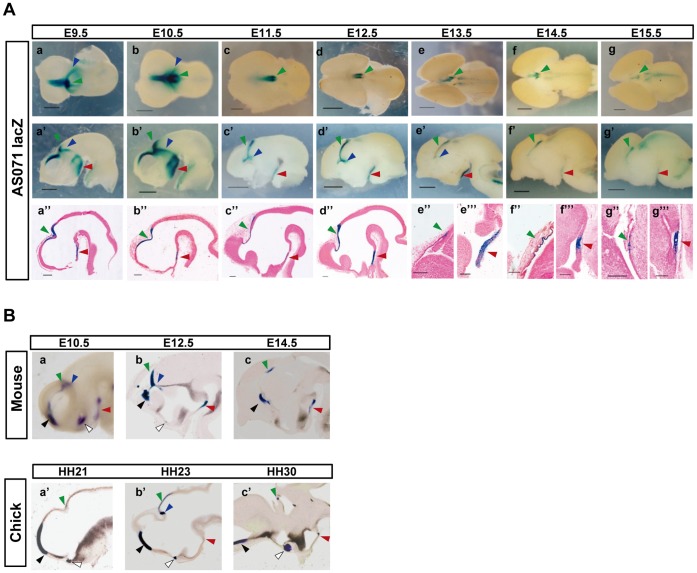
*LacZ* expression in AS071-transgenic mice and comparison of *Fgf8* expression between mouse and chicken. (A) *LacZ* expression pattern in developing mouse diencephalon directed by AS071 in the transgenic Line C. X-Gal staining for β-galactosidase activity in AS071-*lacZ* transgenic mouse embryos through E9.5–E15.5 shows a dynamic pattern of *lacZ* expression in the diencephalic domain. An ectopic expression in midbrain peculiar to this line was observed at E15.5. (a–g) Dorso-frontal views of whole-mount stained dissected brains. (a’–g’) Lateral views. The left telencephalon was removed. (a”–g”, e”’–g”’) Sagittal sections. The expression profiling of all three lines is shown in Figure S2. Scale bars: 0.5 mm (a–c, a’–c’), 1.0 mm (d–g, d’–g’), 0.4 mm (a”–g”, e”’–g”’). (B) Comparison of spatiotemporal *Fgf8* expression between mouse and chicken by in situ hybridization. The mouse E10.5, E12.5 and E14.5 stages correspond to chicken HH21, HH23 and HH30, respectively. Colored arrowheads indicate as follows: green, dorsal midline of the diencephalon; blue, lateral wall of the diencephalon; red, ventral midline of the hypothalamus; black, anterior neural ridge; white, optic recess.

To compare the spatiotemporal enhancer function of AS071 with the expression pattern of *Fgf8* in more detail, we established three stable lines of transgenic mice (Line A, B, and C) bearing a *lacZ* reporter gene construct containing the mouse AS071 (586 bp, Chr19∶45,641,837–45,642,422). *LacZ* expression in F1 or F2 heterozygous embryos was examined at various developmental stages and compared with the *Fgf8* expression pattern in the developing diencephalon. Figure 2A shows the *lacZ* expression profiling of Line C. Consistent *lacZ* expression was observed between E9.5 and E15.5 in three distinct diencephalic domains among the three independent stable lines (Figure 2A, Figure S2): the dorsal midline of the diencephalon extending from the caudal telencephalon (DD, green arrowhead), a narrow band stretch the lateral wall of the diencephalon from dorsal midline (LD, blue arrowhead), and the ventral midline of the hypothalamus (VMH, red arrowhead). This expression pattern is identical to our previous observations in transient AS071-transgenic embryos at E10.5 and E11.5 [24]. Because no consistent *lacZ* expression was observed among the three transgenic lines at E8.5 (Figure S2), the onset of the enhancer activity probably occurs between E8.5 and E9.5.

The *lacZ* expression in all the three domains continued at high levels until E10.5 (Figure 2A-b, b’, b”). By E11.5, the expression in DD extended more caudally in the diencephalon while that in the telencephalon was reduced, and the diffuse expression in hypothalamus was restricted to VMH, and the expression in LD remained, although it became weaker (Figure 2A-c, c’, c”). At E12.5, the *lacZ* expression in DD extended more caudally and reached into the prospective pineal gland, and the narrow band of expression in VMH was also maintained (Figure 2A-d, d’, d”). At E15.5, the expression in DD became much weaker but still remained in the restricted domain surrounding the pineal gland, and the expression in VMH also remained (Figure 2A-g, g’, g”, g”’). The expression pattern in the transgenic embryos was generally consistent among the three independent stable lines though there were some ectopic expressions peculiar to each line. The expression profiling of all three lines is shown in Figure S2.

Comparison of expression patterns of AS071-directed *lacZ* (Figure 2A) with that of *Fgf8* (Figure 2B) revealed a striking resemblance–both spatially and temporally–in the mouse diencephalon (Figure 2A-b’, d’, f’, Figure 2B-a, b, c). This result strongly supported our previous findings that AS071 acts as a distal enhancer for *Fgf8* expression in the developing diencephalon.

### Diencephalic *Fgf8* Expression is Enhanced by AS071 in Mammals

We previously showed a difference in *Fgf8* expression pattern of diencephalic *Fgf8* between mouse at E10.5 and chicken at the corresponding stage [24], [38]. Here, we further compared *Fgf8* expression at three stages of mouse development, namely E10.5, E12.5 and E14.5, each of which corresponds to chick Hamburger-Hamilton (HH) stages 21, 23, and 30. Indeed, the spatial and temporal expression pattern of *Fgf8* in the diencephalon strikingly differed between mouse and chicken, indicating the significant difference in total diencephalic *Fgf8* level in these two species (Figure 2B). In particular, mouse *Fgf8* was strongly expressed as early as E10.5 in DD, LD, and VMH as well as the anterior neural ridge in telencephalon (black arrowhead) and optic recess at the border of telencephalon and diencephalon (white arrowhead). In chicken, however, the expression in the diencephalic domains was not detected at the corresponding developmental stage HH21 except for weak expression in LD. Also, in chicken, the *Fgf8* expression in DD and VMH was observed at HH23, corresponding to mouse E12.5. Especially, in mouse, *Fgf8* expression in VMH was maintained at a high level at least until E14.5, whereas that in chicken was restricted to a small domain throughout its expression period. Consequently, the early onset and the continuous high-level *Fgf8* expression resulted in an increased total amount of *Fgf8* in the mouse diencephalon. It should be noted that the *Fgf8* expression level did not differ significantly between mouse and chick in other domains such as the anterior neural ridge, most prominent expression domains known as a signaling center. These findings provided additional support for the idea that diencephalic *Fgf8* expression is enhanced by AS071 and that the increased *Fgf8* level might be involved in the formation of mammalian-specific features of the brain.

### Functional Dissection of AS071 Reveals Three Distinct Sub-elements

**Figure 3:**
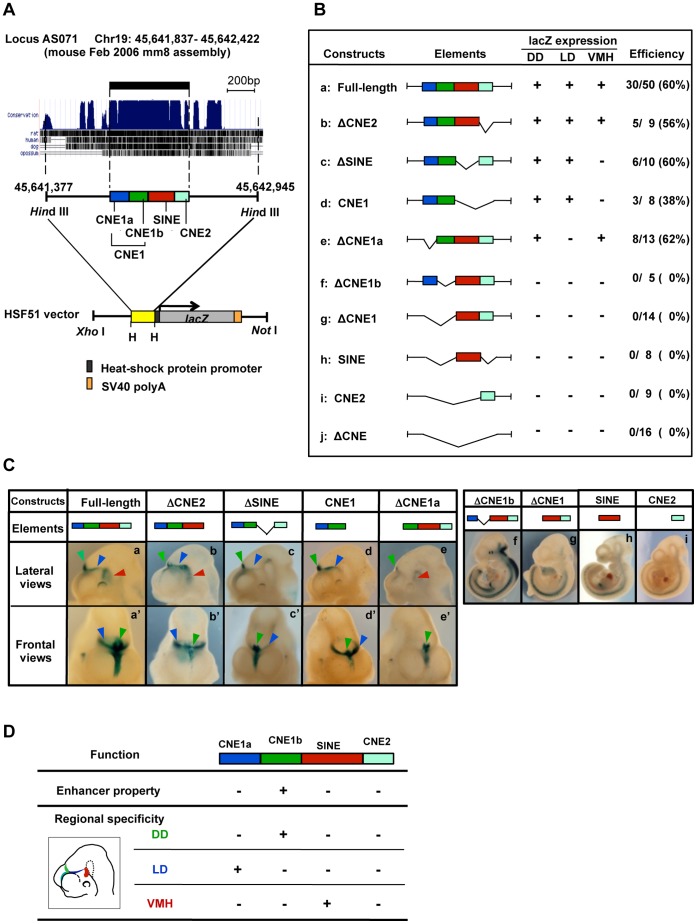
Functional dissection of AS071 using deletion constructs. (A) Genomic region surrounding the mouse AS071 in the UCSC Genome Browser (chr19∶45,641,837–45,642,422, NCBI37/mm9) and schematic representation of the transgene constructs. AS071 was split into four sub-elements for the deletion analyses: CNE1a, blue box; CNE1b, green box; SINE, red box; CNE2, turquoise box. The fragments with various combinations of AS071 sub-elements were amplified from the AS071-HSF51 construct and re-introduced in the HSF51 vector harboring the mouse heat-shock protein promoter (black box) and bacterial *lacZ* reporter gene (grey box) followed by the SV40 polyA signal (orange box). Resulting constructs were linearized with *Xho* I and *Not* I before microinjection. (B) Results of the enhancer analysis using the AS071-deletion constructs. The letters a–j on the left correspond to those in (C). Schematic diagrams of the organization of each construct are shown. The presence (+) or absence (–) of *lacZ* expression in each expression domain is indicated. Efficiency denotes the number of embryos showing *lacZ* expression per those carrying the transgene. (C) (Left) *LacZ* expressions directed in the diencephalic region by the deletion constructs. Note that all constructs lacking the SINE sub-element (red box) could not direct *lacZ* expression in the ventral midline of the hypothalamus. Arrowheads indicate as follows: green, dorsal midline of the diencephalon; blue, lateral wall of the diencephalon; red, the ventral midline of the hypothalamus. (Right) The constructs lacking CNE1b (green box) show no enhancer activity in the diencephalon. (D) Summary of the functional dissection of AS071. Whether each AS071-enhancer sub-element showed activity in a particular region is indicated by + or –. The color of each element corresponds to the associated diencephalic domain shown in the illustration. DD, dorsal midline of the diencephalon; LD, lateral wall of the diencephalon; VMH, ventral midline of the hypothalamus.

To determine the core sequence essential for AS071 activity and the role of the SINE-derived element, we conducted an enhancer analysis with transient transgenic mice using deletion constructs lacking various regions of AS071. We designed the deletion constructs by splitting the AS071 sequence into three sub-elements (Figure 3A, Figure S1): CNE1 (266 bp), SINE (203 bp), and CNE2 (117 bp). The SINE is an AmnSINE1-derived element, i.e., able to be aligned with the consensus sequence of AmnSINE1. For later experiments, CNE1 was further divided into near-halves, i.e., CNE1a (120 bp) and CNE1b (146 bp).

A series of the deletion constructs with various combinations of each AS071 sub-element as well as the flanking sequence were then tested for enhancer activity by means of whole-embryo staining for *lacZ* activity at E10.5 (Figure 3B, C). Deletion of the entire AS071 sequence (ΔAS071) abolished enhancer activity, indicating that the enhancer activity is driven by AS071 (Figure 3B-j). Interestingly, constructs lacking the SINE sub-element (ΔSINE) failed to drive *lacZ* expression only in VMH (Figure 3B-c, and Figure 3C-c, c’). Notably, a construct carrying only the SINE sub-element could not direct *lacZ* expression in any diencephalic domain (Figure 3B-h and Figure 3C-h, h’). These results indicated that the SINE sub-element plays a role only in directing AS071 activity in VMH.

On the other hand, CNE1 sub-element alone could drive *lacZ* expression in the DD and LD (Figure 3B-d and Figure 3C-d, d’), suggesting that the core sequence of the AS071resides within CNE1 sub-element. To further narrow down the core sequence, two deletion constructs, one lacking CNE1a and one lacking CNE1b, were subjected to further enhancer analysis. Exclusion of CNE1a sub-element (ΔCNE1a) resulted in loss of *lacZ* expression in LD, whereas expression in DD and VMH were maintained (Figure 3B-e and Figure 3C-e, e’). Exclusion of CNE1b sub-element (ΔCNE1b), however, abrogated *lacZ* expression in all three domains (Figure 3B-f and Figure 3C-f, f’), consistent with results for other constructs lacking CNE1b sub-element (ΔCNE1, SINE, CNE2, Figure 3B-g, h, i and Figure 3C-g, h, i). These data indicated that the core sequence essential for the AS071 resides within the CNE1b sub-element and that CNE1b sub-element also contains the sequence that directs AS071 activity to DD. CNE1a sub-element appears to contribute only to specifying AS071 activity in LD.

Deletion of CNE2 sub-element (ΔCNE2) did not substantially affect the enhancer activity (Figure 3B-b and Figure 3C-b, b’). As expected, the CNE2 sub-element alone failed to drive any lacZ expression (Figure 3B-j and Figure 3C-j, j’). However, expression in the caudal midline of the telencephalon was slightly reduced in two of the CNE2-lacking constructs (ΔCNE2 and CNE1), suggesting a possible involvement of CNE2 sub-element in enhancer activity in this domain or stabilization of the AS071 activity (Figure 3B-b, b’ and Figure 3C-d, d’).

Figure 3D summarizes the data of the enhancer assay using transient transgenic mice. Although our extensive enhancer assay with various deletion constructs showed that AS071 can be divided into three functional sub-elements, it should be noted that these data were obtained from transient transgenic embryos only at E10.5.

### The SINE-derived Element Determines Domain-specificity of AS071 Activity to VMH

An enhancer generally controls spatiotemporal gene expression, and its activity potentially involves the determination of both the expression domains and timing of expression. To determine whether the SINE sub-element is also involved in the temporal enhancer activity of AS071, we examined whether the lack of enhancer activity in VMH upon deletion of the SINE sub-element was only a transient effect at E10.5, i.e., the SINE sub-element may contribute only to determining the timing of enhancer activity rather than to specify enhancer activity to VMH. Accordingly, we established three lines of the AS071-ΔSINE-stable transgenic mice (Line D, E, and F) carrying the AS071 construct lacking the SINE sub-element (AS071-ΔSINE-HSF51) and compared the spatiotemporal *lacZ* expression in AS071-ΔSINE-transgenic embryos with that in AS071-transgenic embryos.

**Figure 4:**
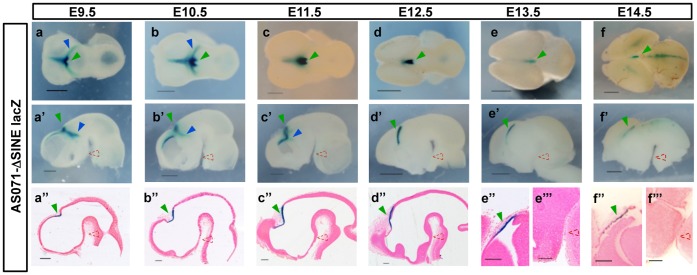
Comparison of spatiotemporal *lacZ* expression pattern between the AS071-ΔSINE-and AS071-transgenic lines. *LacZ* expression in AS071-ΔSINE-transgenic embryos shows exactly same pattern as that in AS071-transgenic embryos between E9.5–E14.5 except in the ventral midline of the hypothalamus (red broken-line triangle), indicating the role of the SINE sub-element in determining domain-specificity of the activity to the ventral midline of the hypothalamus. (a–f) Dorso-frontal views of whole-mount stained dissected brains. (a’–f’) Lateral views. The left telencephalon was removed. (a”–f”, e”’–f”’) Sagittal sections. Colored arrowheads represent as follows: green, dorsal midline of the diencephalon; blue, lateral wall of the diencephalon; red, the ventral midline of the hypothalamus. Scale bars: 0.5 mm (a–c, a’–c’), 1.0 mm (d–g, d’–g’), 0.4 mm (a”–g”, e”’–g”’).

As shown in Figure 4, in the AS071-ΔSINE-transgenic embryos (Line D), the *lacZ* expression disappeared only in VMH, whereas the expressions in DD and LD were the same between AS071-and AS071-ΔSINE-transgenic embryos (Figure 2A, Figure 4). This observation was consistent with results from the transient deletion assay (Figure 3B-c, C-c, c’). Furthermore, in AS071-ΔSINE-transgenic embryos, *lacZ* expression in VMH was not observed at any other later developmental stages, whereas expression in DD and LD was the same as that in AS071-transgenic embryos through E9.5–E14.5 (Figure 4). This *lacZ* expression pattern was consistent among the three independent stable lines (Figure S3). These observations indicated that the SINE sub-element contributes to enhancer activity of AS071 by specifying activity to VMH rather than by changing the timing of expression.

### Possible Transcription Factors that Associate with AS071

To elucidate the molecular basis of *Fgf8* regulation by AS071, we attempted to identify possible molecules that bind to the AS071 sequence. Based on a database of putative transcription factor binding sites (TFBSs), three TFBSs for POU3F2, NR3C1, and HMX1 were tested in the deletion assay (Figure S4). The complete deletion of all three TFBSs (45 bp of the 586 bp of the AS071 sequence) did not significantly alter the *lacZ* expression pattern in E10.5 embryos (data not shown). We noticed, however, that the edge of its expression domain was not clear compared to that directed by the full-length AS071 construct. This may simply have been a consequence of changing the spacing between the shortened DNA sequence and possible transcription factors responsible for the enhancer activity. However, it is possible that any of these three transcription factors are involved in AS071. Point mutations that disrupt the binding motifs will be necessary to confirm this issue.

## Discussion

### AS071 is a Distal Enhancer for Mammalian *Fgf8* Expressed in the Developing Diencephalon

Using an enhancer assay with transient transgenic mice, we previously reported that the *lacZ* expression directed by AS071 resembles that of *Fgf8* in the developing diencephalon at specific developmental stages (E10.5 and E11.5). In the current study, by making three independent AS071-transgenic lines (Figure 2A and Figure S2), we analyzed the expression of *lacZ* in transgenic embryos from developmental stages from E8.5 to E15.5 and obtained unambiguous data, in which the expression pattern of *lacZ* driven by AS071 recapitulates that of diencephalic *Fgf8*, confirming our previous findings. Although several enhancer elements have been reported that regulate *Fgf8* expression in many tissues, including brain region ([39] see below), this is the first demonstration of a mammalian *Fgf8* enhancer specific for its diencephalic expression.

*Fgf8* is expressed in the diencephalic domain during embryogenesis of mouse, chicken, and zebrafish [26], [27], [40]. This suggests that *Fgf8* plays critical roles in vertebrate development and that the regulatory elements governing diencephalic *Fgf8* expression are expected to be conserved during vertebrate evolution. Although we already described that there is no sequence homologous to AS071 at the syntenic position of the chicken genome, namely in the intronic region of the gene *RP11-529110.4*, we investigated whether there might be sequences similar to AS071 in the chicken genome, even in non-homologous regions. We did not, however, find any such sequence in the chicken genome. This indicates that AS071 was newly acquired in the genome of a mammalian common ancestor and that the diencephalic expression of *Fgf8* in vertebrates other than mammals is regulated by their own diencephalic *Fgf8* enhancer. As described in the Results section, although the expression domain of *Fgf8* is essentially the same, we observed differences in both the onset and level of diencephalic *Fgf8* expression between mouse and chicken (Figure 2B). This difference might be explained by the presence of AS071. Whether AS071 is a unique enhancer responsible for the diencephalic expression of *Fgf8* in mammals or plays a role in addition to already existing enhancers remains to be determined.

### Origin of the Three Functionally Distinct Sub-elements within AS071

Recent reports have shown that developmental genes are often regulated by multiple discrete enhancer modules with wholly or partially overlapped activities, redundancies of which can be explained based on the need for robustness of gene expression against adverse environmental conditions (such as shadow enhancers) [41]–[43]. In other cases, a single enhancer may drive activity in multiple tissues [44] or at different developmental stages [45]. Such a multifunctional enhancer may be comprised of several sub-elements having different activities. The structure of such a multifunctional enhancer, however, has not been sufficiently examined in detail with regard to the functions of respective sub-elements.

**Figure 5:**
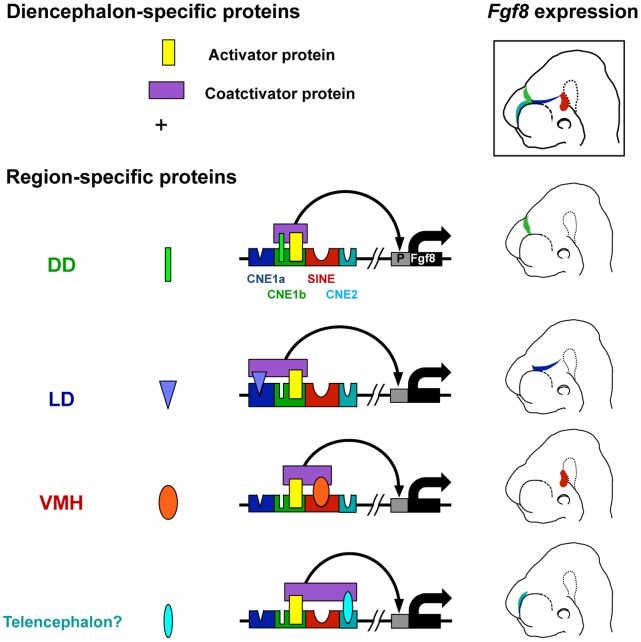
A model of domain-specific expression of diencephalic *Fgf8* by AS071. Spatiotemporally restricted *Fgf8* expression in the developing diencephalon by AS071 is accomplished through the interaction of activator proteins that cooperatively bind to each sub-element. The coactivator protein (purple box) mediates the interaction between the activator protein (yellow box), which is ubiquitously expressed in the diencephalon, and other domain-specific activator proteins, as indicated by the colored shapes. Each domain-specific activator protein needs to associate with the core element in CNE1b to effect enhancer activity in each diencephalic domain.

Our enhancer analysis using a series of deletion constructs with various combinations of each AS071 sub-element revealed that AS071 consists of at least three functionally distinct sub-elements, i.e., AS071 functions as an enhancer unit via orchestrated regulation by the three sub-elements with respective domain-specificities for activity, including one core sub-element essential for overall enhancer function. This single-core enhancer unit can direct the activity to all three diencephalic expression domains. From this viewpoint, AS071 exhibits a unique aspect of gene regulation. Thus, these findings allow us to propose a model for the regulatory mechanism of AS071 (Figure 5). The domain-specific *Fgf8* expression in the developing diencephalon by AS071 is accomplished through the interaction of multiple activator proteins that cooperatively bind to each sub-element. The activator protein and the coactivator protein both are ubiquitously expressed throughout the diencephalon and thus are shared in all cases. The coactivator protein mediates the interaction between the diencephalon-specific activator protein essential for the display of enhancer activity and other domain-specific activator proteins that specify enhancer activity to each domain. Thereby, the enhancer activity in each diencephalic domain is invoked.

It will be interesting to determine how the multiple sub-elements of AS071 have acquired their functions and came to be conserved as a mammalian-specific CNE during evolution. Because it is unlikely that multiple mutations that provide advantages for host survival occurred simultaneously at individual sites of each sub-element, it is reasonable to assume that each sub-element within AS071 underwent an independent but sequential acquisition of function. From the fact that CNE1b sub-element contains the core sequence essential for AS071 activity, the following evolutionary scenario of AS071 can be deduced. In the genome of a mammalian common ancestor, CNE1b sub-element initially acquired the enhancer activity for *Fgf8* expression in the dorsal midline of the diencephalon and was conserved as a CNE. Subsequently, the two flanking sequences of CNE1b sub-element, namely AmnSINE1 and present-day CNE1a sub-element, might progressively have acquired their functions to serve an additional domain-specificity such as VMH and LD, respectively. This organizational feature of AS071 composed of multiple sub-elements allows one enhancer unit to produce the spatiotemporally complex patterns of the *Fgf8* expression by means of sharing one core element. This may be an alternative strategy to add a new function to the existing enhancer, leading to the generation of extra domains and/or a change in levels of *Fgf8* expression. As a result of coevolution of the multiple functional sequences, AS071 was established as an enhancer unit in the genome of a mammalian common ancestor and provided a high level of *Fgf8* expression in the mammalian diencephalon.

### The SINE Sub-element in AS071 is Involved only in Determining Domain-specificity to VMH

The comprehensive expression profiling of the AS071-ΔSINE-transgenic mice revealed that the SINE sub-element contributes to specify enhancer activity to VMH. It should be noted that this domain-specificity is highly restricted to VMH because the other expression domains, DD and LD, were not affected by the deletion of the SINE sub-element. This is the first example of an enhancer that can be experimentally divided into distinct sub-elements with different functions, namely the enhancement of activity and the determination of domain-specificity.

The domain-specific expression of developmental genes is considered to be determined by the combination of trans-acting factors expressed in a particular tissue. In the present case, there may be trans-acting-factors–specifically expressed in VMH that bind to the SINE sub-element to ensure the hypothalamic expression of *Fgf8*, as explained in the previous section. We investigated possible sequence motifs for binding of known trans-acting factors specifically expressed in hypothalamus in the SINE sequence. This quest, however, was unsuccessful.

Furthermore, comparison of the SINE sub-element of the AS071-enhancer and the zebrafish *Fgf8* enhancer, *fgf.dr18*, which directs *Fgf8* expression to the head region including Hypo [40], revealed no sequence similarity or shared motifs between these two hypothalamic *Fgf8* enhancers (data not shown).

Franchini et al. characterized two mammalian hypothalamic enhancers of the proopiomelanocortin gene (*POMC*), nPE1 and nPE2 [25]. These enhancers drive similar *POMC* expression patterns in the arcuate nucleus in mouse hypothalamus and share three nucleotide motifs and eight putative TFBSs [22], although they originated from different retroposons, namely an MaLR retroposon and an MIR SINE, respectively. Accordingly, Franchini et al. concluded that the enhancers are the consequence of convergent molecular evolution of two unrelated retroposons [22]. Because the AS071 SINE sub-element also participates in determining the hypothalamic specificity of the enhancer activity, even though the SINE sub-element alone is incapable of driving activity, we performed a comparative motif analysis among three retroposon-derived hypothalamic enhancers. We found three de novo nucleotide motifs shared among nPE1, nPE2 and AS071 (Figure S5). In focusing on the AS071 SINE sub-element involved in the hypothalamic enhancer activity, one of the motifs (Motif 1) present in the AS071 SINE sub-element is shared with nPE1 and nPE2. In addition, Motif 2 is shared between the SINE sub-element and nPE1. Whether these motifs are actually responsible for determining domain-specificity to hypothalamus remains to be examined.

### Possible Implications of Diencephalic *Fgf8* in Mammalian-specific Brain Formation

As described in the Results section, the level of diencephalic *Fgf8* expression seems to be enhanced specifically in mammals even though the expression domains are fundamentally conserved across the vertebrate species (Figure 2B). We previously demonstrated that the Fgf8 signaling in LD is required for the proper patterning of thalamic nuclei [46], and suggested that the enhanced *Fgf8* level in diencephalon might be involved in the formation of mammalian brain. This led us to propose a hypothesis in which AS071 is involved in the generation of a new thalamo-cortical somato-sensory system through whisker due to nocturnal life of primordial mammals [11]. To date, the significance of *Fgf8* signaling in the development of diencephalon has not been sufficiently analyzed. Several studies using the *Fgf8* hypomorphic mice, however, provided some implications about the involvement of enhanced diencephalic Fgf8 signaling in mammalian-specific brain formation.

Brooks et al. discovered a significant reduction of oxytocin-containing neurons in the hypoththalamic nuclei of homozygous *Fgf8* hypomorphic mice [47], and suggested that the Fgf8 signaling participates in the maturation of the oxytocin system that originates in the diencephalon [47]. Traditionally, oxytocin, a mammalian endocrinal hormone, is involved in maternal functions such as regulating uterine contractions during labor and modulating milk ejection [48]. In addition, recent studies have provided evidence that the neurohormonal action of oxytocin is associated with various important behaviors in mammals including maternal behaviors, social recognitions, and pair-bonding [49]–[51]. The maintenance of harmonious social relationships is necessary for mammals whose life span is long, and in addition, a robust bond of mother and children or mate pair would increase offspring survival. The involvement of Fgf8 signaling may contribute to the establishment of mammalian social organization through the actions of oxytocin.

In *Fgf8* hypomorphic mice, it is also demonstrated that the size of a pineal gland was reduced corresponding to the decreasing Fgf8 activity in DD [52]. The pineal grand plays a crucial role in regulating the circadian rhythms in most non-mammalian vertebrates through both photo input and melatonin output. However, in the case of mammals, a pineal gland is specialized to synthesize and release melatonin under the regulation through the suprachiasmatic nuclei (SCN) of the hypothalamus, the primary pacemaker of the circadian rhythms [53], [54]. This evolutionary trend corresponds to dramatic changes in the structure and function of the pineal gland, i.e., the mammalian pinealocytes have lost the ability to respond to light whereas the photoreceptor cells in the pineal gland of non-mammalian vertebrates can function as a light detector [55], [56]. Enhanced Fgf8 activity in DD might have contributed to the alteration of cell fate in the mammalian pineal gland. The evolution of this new system to catch the modicum of light in the dark might have been needed to keep the body clock constantly fine-tuned for early nocturnal mammals [11]. A suggestion that the *Fgf8* expressed in the regions surrounding the optic chiasma may participate in the development of hypothalamic nuclei including SCN [57] supports this idea.

It must be noted that the overall Fgf8 activity, not only in the diencephalon, is reduced in *Fgf8* hypomorphic mice. Therefore, these morphological defects observed in *Fgf8* hypomorphic mice are not necessarily due to the reduction of the diencephalic fgf8 activity. Further understanding about the role of Fgf8 activity in development of diencephalon would reveal the contribution of enhanced *Fgf8* expression to generating the mammalian-specific traits.

### Contribution of Retroposons to Genomic Evolution

Owing to their repetitive nature, the biggest impact of retroposons on genome structure is to increase non-coding regions. Because a certain amount of mammalian CNEs are derived from retroposons (see Introduction), they may provide plasticity to genomic structure. The repetitive nature of retroposons would become more important if originally the retroposons contained motifs that could be bound by certain trans-acting proteins. For example, the MER20 transposons are frequently observed adjacent to genes expressed in endometrial cells [58]. The authors proposed that MER20 sequences have been used as various expression regulatory elements such as insulators and have provided a novel gene regulatory network involved in pregnancy in placental mammals. In addition, Schmidt et al. identified thousands of regions that bind the transcriptional repressor, CTCF, and these regions were derived from retroposons in six representative mammals, suggesting that retroposons have been an important source of insulators during mammalian evolution [59]. These findings support a model in which a retroposon carrying a certain functional sequence(s) can be a source of dispersed regulatory DNA elements and produce a novel regulatory network for gene expression [4] [60].

We previously described that an AmnSINE1 locus, *AS021*, contains several binding motifs for known transcription factors, such as Oct-1 and Brn-1, which are also shared by multiple AmnSINE1 copies [25]. This fact may support the notion that AmnSINE1s contained such primordial motifs when the original AmnSINE1 sequences were amplified in a common ancestor of mammals and that a new regulatory network system mediated by AmnSINE1s contributed to the acquisition of mammalian-specific traits.

In contrast with *AS021*, we did not find any known shared motifs in the SINE-derived sub-element of AS071, although the similarity between the consensus sequence of AmnSINE1 and the SINE-derived sub-element of AS071 is relatively high. Accordingly, in the case of AS071, the genomic function was not acquired apparently by taking advantage of the motifs contained in the original sequence. This indicates that there can be multiple ways to become an enhancer from one original sequence. Given the fact that more than 100 AmnSINE1-derived CNEs exist in mammalian genome, investigation of them would be significant to reveal various evolutionary processes of *cis*-regulatory elements.

## Materials and Methods

### Ethics Statement

Mouse strains of B6C3F1, C57BL/6 and ICR used in this study were purchased from Sankyo Laboratory Service Corporation (Tokyo, Japan). Animals were kept in ventilated cages under a 12-h light/dark cycle at room temperature. This study was approved by the Ethics Committee of Tokyo Institute of Technology.

### Transgene Construction

The AS071-HSF51 construct (corresponding to the AS071-HSF51-2 construct in [27]) was made as described [24]. Briefly, a 1.6-kbp fragment (chr19∶45,641,377–45,642,945; mm9) containing 586 bp of AS071 was amplified by PCR from MCH mouse (CLEA Japan, Inc.) genomic DNA using primers AS071-F2 and AS071-R2 (Table S1) and cloned into the HSF51 vector harboring the mouse heat-shock protein 68 promoter (hspp) followed by the bacterial *lacZ* reporter gene [61] and the SV40 poly-A signal through the *Hin*d III sites. The 586 bp of AS071 (chr19∶45,641,837–45,642,422; mm9) was amplified from the AS071-HSF51 construct using primers AS071-F1 and AS071-R1 (Table S1) to generate the AS071-CNE-HSF51 construct (corresponding to AS071-HSF51-1 in [27]).

The deletion constructs containing combinations of the various AS071 sub-elements were generated by overlap extension PCR. Briefly, internal primers of overlapping complementary sequence (Table S1) were designed to carry out the successive deletion of each AS071 sub-element. The first PCR was performed with the internal primer and the vector primer (HSF51-F: 5′-ACCACAGCTGGGTACCGGG-3′ and HSF51-R: 5′-CCGGCTGCTCAGTTTGGAT-3′) using the AS071-HSF51 construct as a template. The resulting two PCR fragments were purified using the Gel Extraction kit (Qiagen) and then used as templates for the second PCR with the vector primers to generate the various deletion fragments. Final products were purified and cloned into the HSF51 vector at *Hin*d III sites. These deletion constructs contained the flanking sequence of AS071 so that each of the AS071 sub-elements retained a physical distance of >524 bp from the hspp. The AS071-TFBSs-deletion construct was generated essentially as the other deletion constructs except a third round of PCR was needed to obtain the final product. Inner primers used are listed in Table S2.

Overlap extension PCR amplification was conducted in 50 µl containing 5 ng of plasmid DNA, 0.3 µM of each primer, 0.2 mM of dNTPs, 1 mM of MgSO4, 5 µl of KOD-Plus-buffer, and 1U of KOD-Plus-Polymerase (TOYOBO, Japan). The cycling program was: 25–30 cycles of denature at 94°C for 15 s, annealing at 60°C for 30 s, and extension at 68°C at 1–2 min.

Each construct was confirmed by sequencing. Sequencing reaction was performed using BigDye® Terminator Cycle Sequencing Kit (Applied Biosystems). After ethanol precipitation, samples were resuspended in HiDi™ Formamid (Applied Biosystems), and sequenced on the Applied Biosystems 3130*x/*Genetic Analyzer.

### Production of Transgenic Mice and Genotyping

Transgenic mice were produced as described [24]. Briefly, the constructs were linearized with *Sca* I (for AS071-CNE-HSF51) or with *Not* I and *Xho* I (for other constructs). After purification using the Qiagen Gel Extraction kit, the DNA fragments were dialyzed against microinjection buffer (5 mM Tris-HCl, 0.1 mM EDTA) at 4°C overnight. Pronuclear microinjection was performed using 10 ng/µl of the DNA solution into a B6C3F1 zygote, and microinjected zygotes were transferred to the oviduct of pseudopregnant ICR females. The AS071-CNE-HSF51 construct and AS071-ΔSINE-HSF51 construct were used to generate stable lines of AS071-transgenic mice.

Genotyping PCR was conducted in 10 µl containing 0.5 µl of DNA solution, 0.25 µM of each primer, 0.2 mM of dNTPs, 1 µl of *Ex Taq* buffer, and 0.05U of *Ex Taq* Polymerase (Takara, Japan). The cycling program was: 30 cycles of denature at 95°C for 30 s, annealing at 72°C for 30 s, and extension at 72°C at 1 min. The genomic DNA was extracted from tail or yolk sac using Direct PCR reagent (VIAGEN BIOTECH Inc.). Following PCR primers were used for identifying the transgenic animals: LZSEQ5, 5′-GCGATTAAGTTGGGTAACGCCA-3′; and HSPP681, 5′-ACGCGATTGGAGAGGATCAC-3′.

### Whole-embryo X-Gal Staining and Histological Analysis of Embryos

Transgene (*lacZ*) expression was quantified by whole-embryo X-Gal staining of F1 or F2 offspring as described [24]. Briefly, dissected embryos (E8.5–E12.5 stages) or brains (for later stages) were fixed for 30 min (E8.5–E11.5) or 1 h (later stages) in PBS containing 1% formaldehyde, 0.1% glutaraldehyde, and 0.05% (v/v) NP40. The fixed embryos or brains were washed twice in PBS, and stained with PBS containing 500 µg/ml X-Gal, 5 mM K_3_Fe(CN)_6_, 5 mM K_4_Fe(CN)_6_, 2 mM MgCl_2_, 0.02% NP40, and 0.01% sodium deoxycholate for more than 3 h at 37°C. The stained embryos or brains were then fixed with 4% paraformaldehyde in PBS overnight at 4°C, rinsed several times with PBS, and permeated with 10% sucrose in 0.1 M phosphate buffer (PB) overnight at 4°C. After incubation with 7.5% gelatin and 10% sucrose in 0.1 M PB (GS-PB) for 30–120 min at 37°C, embryos were embedded in GS-PB for sectioning. Sagittal sectioning was performed on a cryostat (Leica CM 1850), and the sections were counterstained with kernechtrot (Nuclear Fast Red).

### In Situ Hybridization (ISH)

Mouse embryos were prepared from ICR mice. For whole-mount ISH in mouse (E10.5), brains were dissected and then fixed overnight in 4% paraformaldehyde dissolved in DEPC-treated PBS at 4°C. For the section ISH (E12.5 and E14.5), brains were fixed overnight in 30% sucrose with 4% paraformaldehyde and embedded with OCT compound (Tissue-Tek, Sakura, Torrance, CA) and frozen. Sagittal sections were prepared on a Leica sledge microtome at 40 µm and individually mounted on slides. Single-or two-color non-radioactive ISH analysis was performed on mouse embryos (E10.5, E12.5, E14.5) and chicken embryos (HH21, HH23, HH30) to visualize expression of *Fgf8* as described [62] with some modifications [38].

### Identification of Possible TFBSs in AS071

To search for transcription factors that bind to AS071, we referred to the UCSC Genome Browser, which provides predicted TFBSs identified from the conserved sequences from the human/mouse/rat alignment. Three significant (*p*<0.01) binding sites of POU3F2 (GCTGTATGTATTCAT, Chr19∶45,641,936–45,641,950 mm9), NR3C1 (AGAAGAACACAGCATGTCCA, Chr19∶45,642,006–45,642,025 mm9), and HMX1 (CAGGCACTTG, Chr19∶45,642,108–45,642,117 mm9), were chosen for the deletion assays (Figure S4).

### Comparative Motif analysis

The matrix-based MEME algorithm (meme.nbcr.net) was used to identify motifs shared among the three retroposon-derived hypothalamic enhancers, AS071, human nPE1, and nPE2 [22].

## Supporting Information

Figure S1 **Sequence conservation of AS071 among mammals.** Alignment of AS071 loci among 19 mammalian species. Colored boxes above the sequences represent the AS071 sub-elements: CNE1a (blue), CNE1b (green), SINE sub-element (red), and CNE2 (turquoise) (see Figure 3A).
(TIF)

Figure S2 ***LacZ*** **expression profiling of three independent stable AS071-transgenic lines.** Spatiotemporally consistent *lacZ* expression pattern among the three independent stable AS071 lines A, B, and C. No consistent *lacZ* expression is observed at E8.5 (a, a’, a”). Consistent *lacZ* expression is observed in the dorsal midline of the diencephalon (green arrowhead), lateral wall of the diencephalon (blue arrowhead), and the ventral midline of the hypothalamus (red arrowhead) from E9.5 to E15.5. Upper (b–h, b’–h’, b”–h”) and lower (i–o, I’–o’, I”–o”) panels are lateral and dorso-frontal views of the dissected brains, respectively.
(TIF)

Figure S3 ***LacZ*** **expression profiling of three independent stable AS071-ΔSINE-transgenic lines.** Spatiotemporally consistent *lacZ* expression pattern among the three independent stable AS071-ΔSINE Lines D, E, and F. *LacZ* expression is observed only in the dorsal midline of the diencephalon (green arrowhead) and lateral wall of the diencephalon (blue arrowhead). *LacZ* was not expressed in the ventral midline of the hypothalamus from E9.5 to E14.5. Upper (a–f, a’–f’, a”–f”) and lower (g–l, g’–l’, g”–l”) panels are lateral and dorso-frontal views, respectively, of the dissected brains.
(TIF)

Figure S4 **Deletion analysis using Δ-TFBS construct.** Putative TFBSs in the AS071 locus identified by the UCSC Genome Browser. Three TFBSs, POU3F2, NR3C1 (in CNE1a), and HMX1 (in SINE sub-element), with *p*<0.01 were chosen for the deletion assay (red asterisks).
(TIF)

Figure S5 **Comparison of motifs in sequences of AS071 and nPE1 and nPE2 enhancers.** Sequence comparison of human AS071 with the two retroposon-derived human enhancers, nPE1 and nPE2 [22]. Sequence analysis of nPE1, nPE2, and the SINE sub-element within AS071 (indicated by bold characters in the orange box) using the matrix-based algorithm MEME (http://meme.nbcr.net/) revealed three previously unrecognized motifs found in at least in five sites in the enhancers. Motif 1 (shown in red characters) is shared among all three retroposon-derived sequences, whereas motif 2 (blue characters) is detected in the SINE sub-element of AS071 as well as nPE1. Motif 3 (green characters) resides in CNE1b but not in the SINE sub-element.
(TIF)

Table S1 **Inner primers used to generate the AS071-deletion constructs.** A series of AS071-deletion constructs was generated by overlap extension PCR using combinations of inner primers and vector (outer) primers. The length of each final insert fragment is shown in the right column.
(TIF)

Table S2 **Inner primers used to generate the Δ-TFBS constructs.** Δ-TFBS constructs were generated by three-step overlap extension PCR using combinations of inner primers and vector (outer) primers.
(TIF)
