# Supplementary figures and images for: A SINE-Derived Element Constitutes a Unique Modular Enhancer for Mammalian Diencephalic Fgf8
Source: PLoS One. 2012 Aug 24;7(8):e43785. doi: 10.1371/journal.pone.0043785 (PMC3427154; doi:10.1371/journal.pone.0043785)

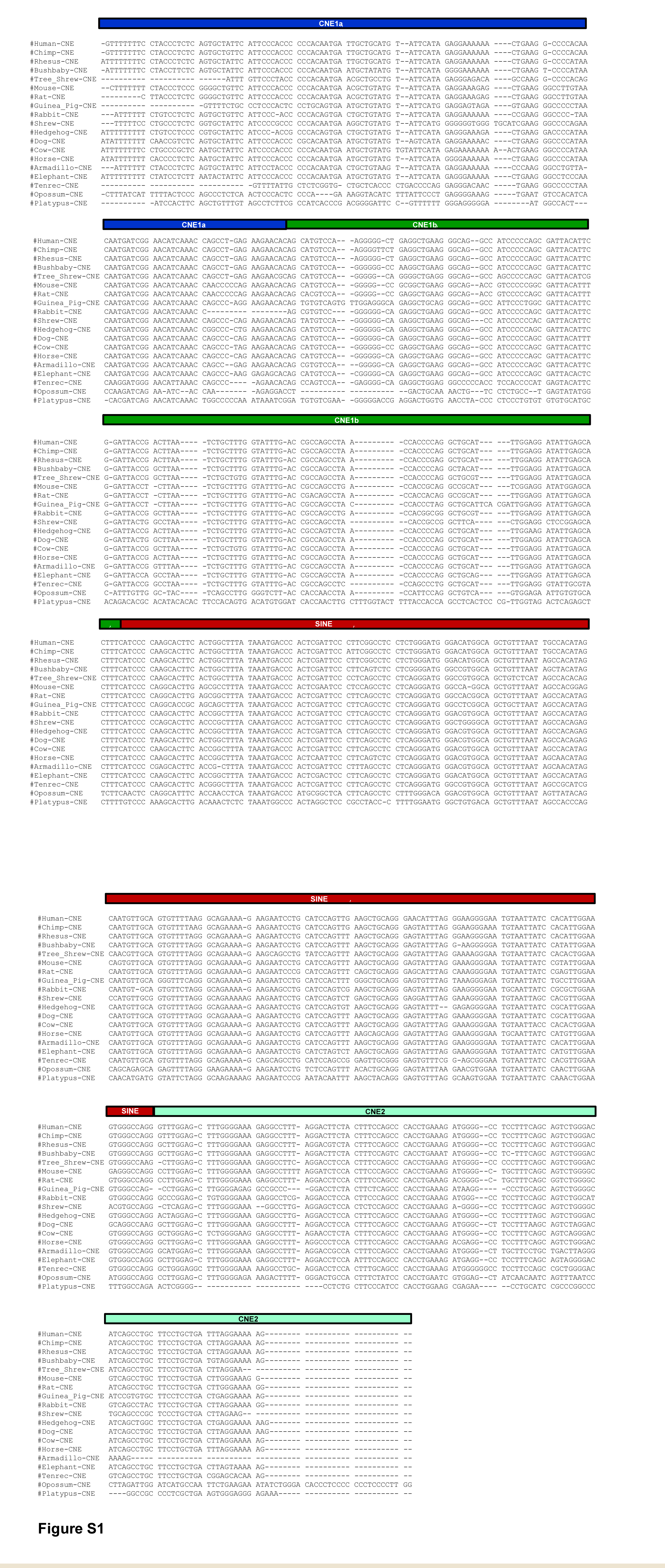

Supplement: Figure S1 — Sequence conservation of AS071 among mammals. Alignment of AS071 loci among 19 mammalian species. Colored boxes above the sequences represent the AS071 sub-elements: CNE1a (blue), CNE1b (green), SINE sub-element (red), and CNE2 (turquoise) (see Figure 3A). (TIF) [file pone.0043785.s001.tif]

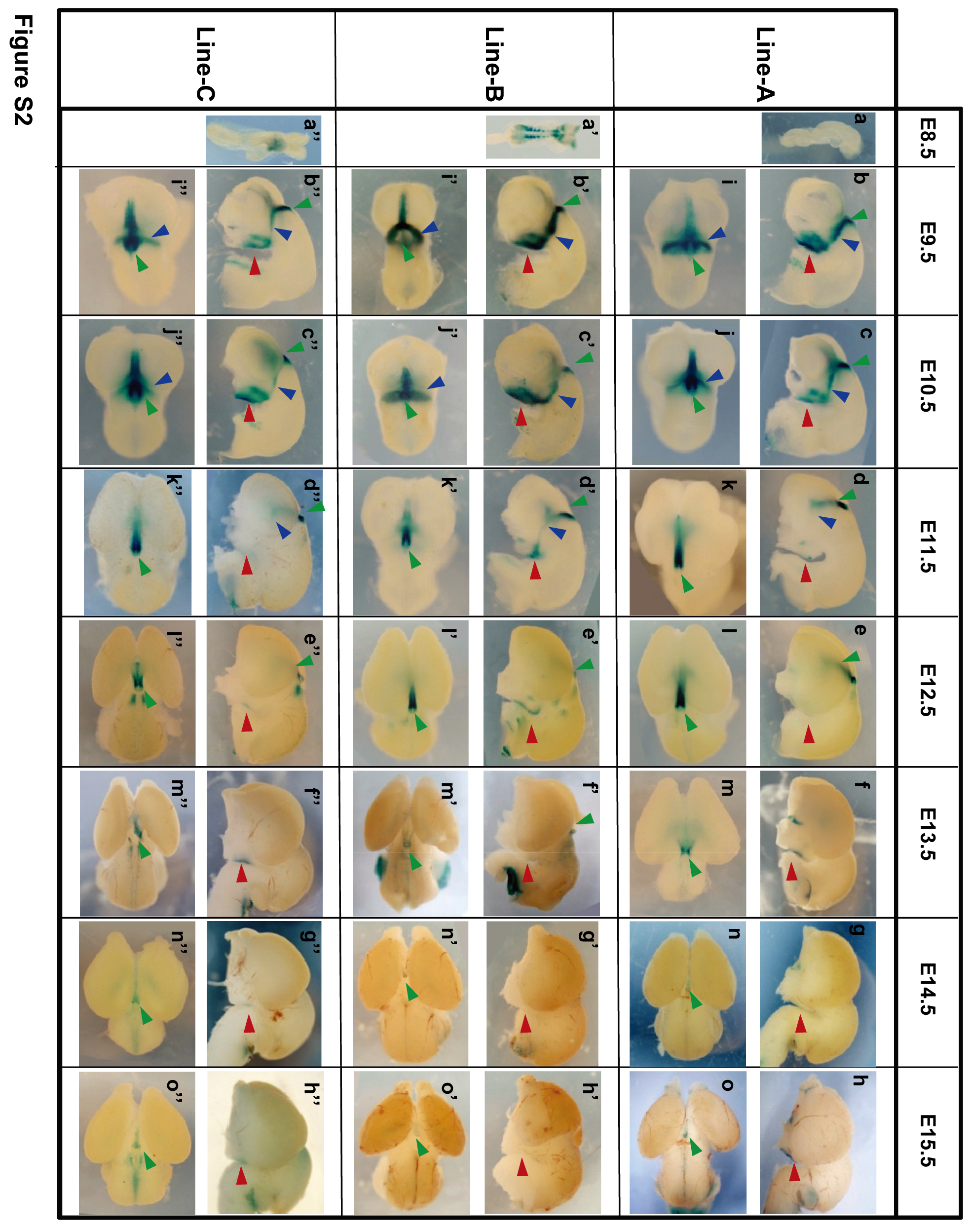

Supplement: Figure S2 — LacZ expression profiling of three independent stable AS071-transgenic lines. Spatiotemporally consistent lacZ expression pattern among the three independent stable AS071 lines A, B, and C. No consistent lacZ expression is observed at E8.5 (a, a’, a”). Consistent lacZ expression is observed in the dorsal midline of the diencephalon (green arrowhead), lateral wall of the diencephalon (blue arrowhead), and the ventral midline of the hypothalamus (red arrowhead) from E9.5 to E15.5. Upper (b–h, b’–h’, b”–h”) and lower (i–o, I’–o’, I”–o”) panels are lateral and dorso-frontal views of the dissected brains, respectively. (TIF) [file pone.0043785.s002.tif]

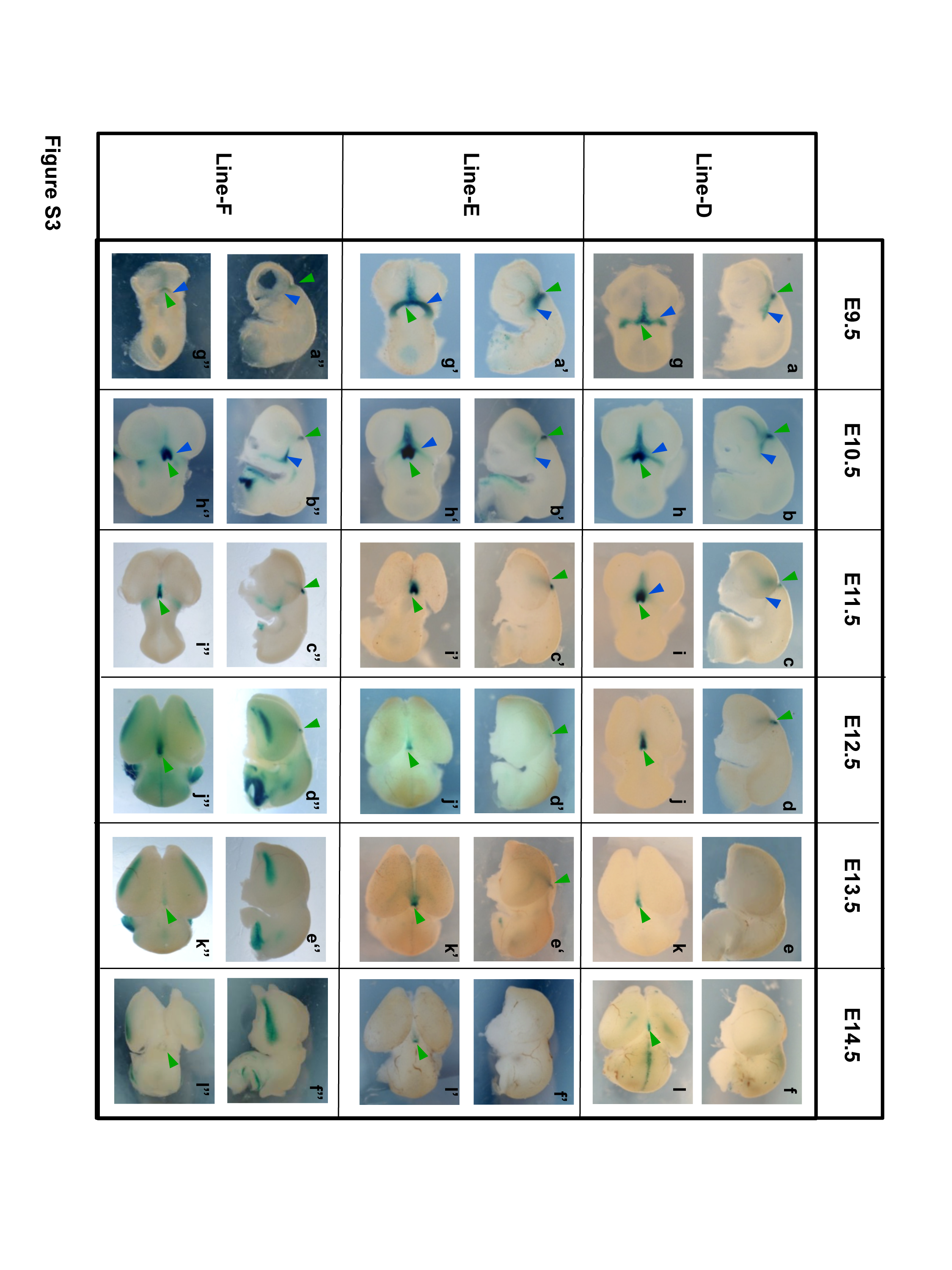

Supplement: Figure S3 — LacZ expression profiling of three independent stable AS071-ΔSINE-transgenic lines. Spatiotemporally consistent lacZ expression pattern among the three independent stable AS071-ΔSINE Lines D, E, and F. LacZ expression is observed only in the dorsal midline of the diencephalon (green arrowhead) and lateral wall of the diencephalon (blue arrowhead). LacZ was not expressed in the ventral midline of the hypothalamus from E9.5 to E14.5. Upper (a–f, a’–f’, a”–f”) and lower (g–l, g’–l’, g”–l”) panels are lateral and dorso-frontal views, respectively, of the dissected brains. (TIF) [file pone.0043785.s003.tif]

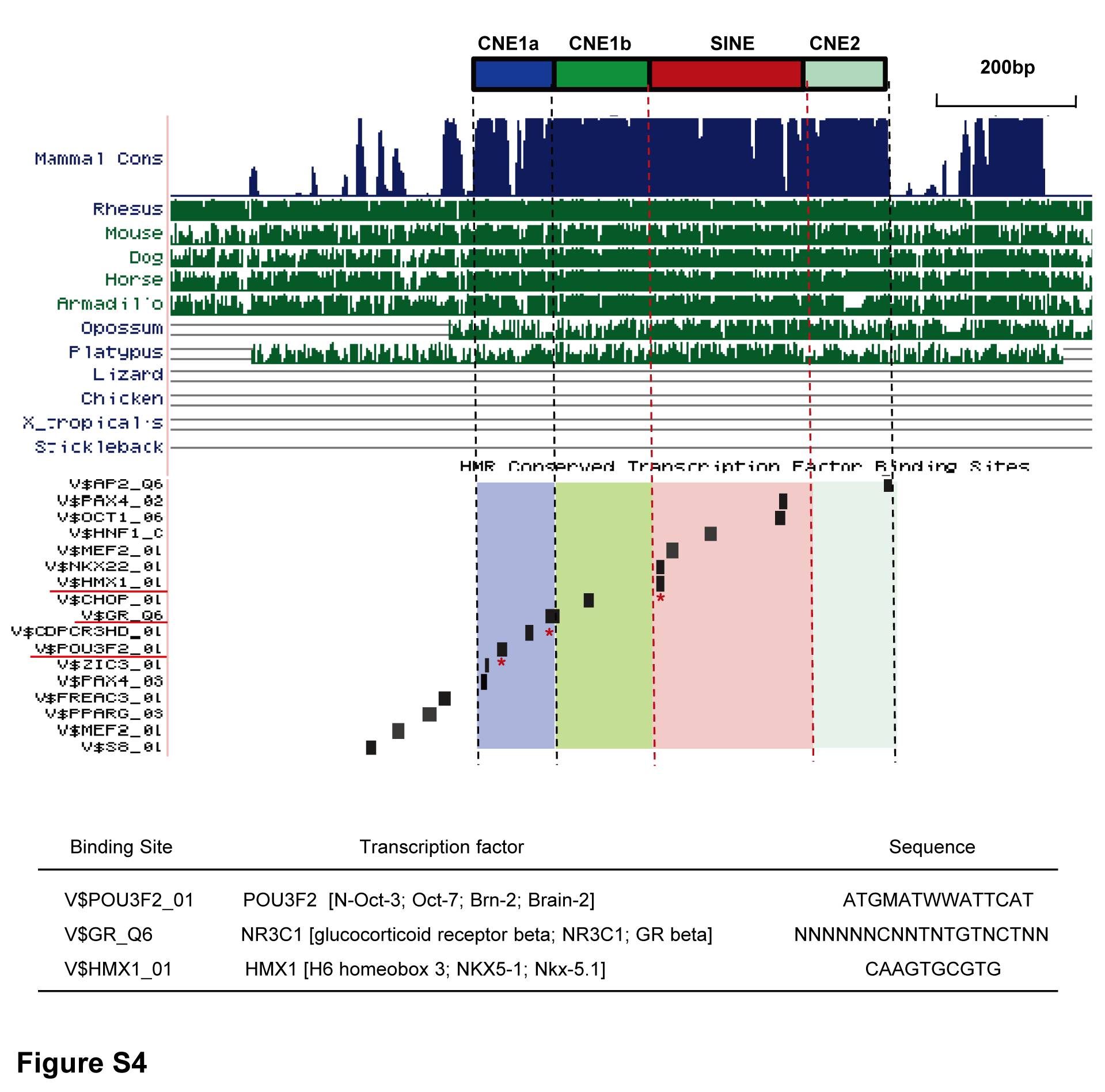

Supplement: Figure S4 — Deletion analysis using Δ-TFBS construct. Putative TFBSs in the AS071 locus identified by the UCSC Genome Browser. Three TFBSs, POU3F2, NR3C1 (in CNE1a), and HMX1 (in SINE sub-element), with p<0.01 were chosen for the deletion assay (red asterisks). (TIF) [file pone.0043785.s004.tif]

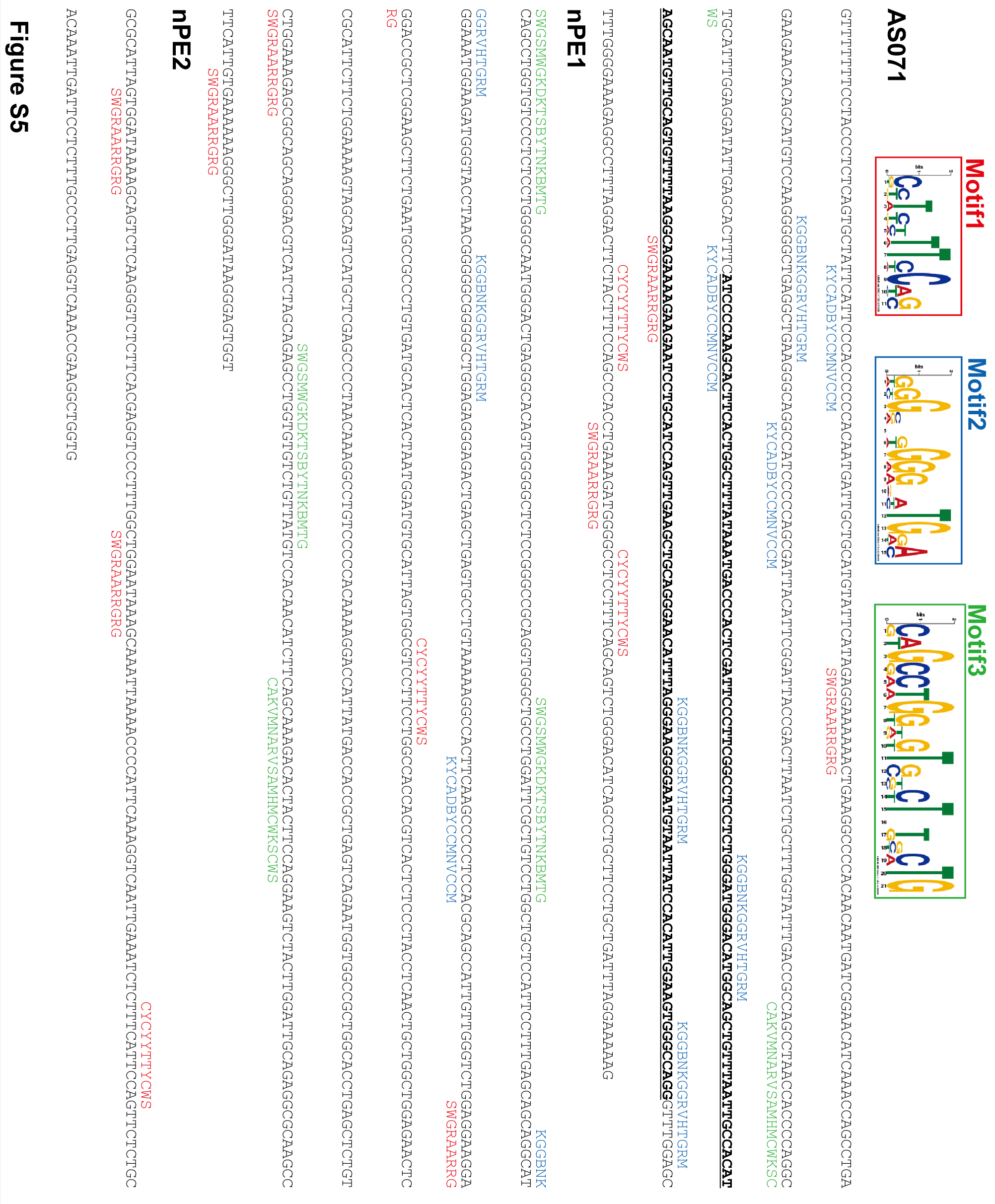

Supplement: Figure S5 — Comparison of motifs in sequences of AS071 and nPE1 and nPE2 enhancers. Sequence comparison of human AS071 with the two retroposon-derived human enhancers, nPE1 and nPE2 [22]. Sequence analysis of nPE1, nPE2, and the SINE sub-element within AS071 (indicated by bold characters in the orange box) using the matrix-based algorithm MEME (http://meme.nbcr.net/) revealed three previously unrecognized motifs found in at least in five sites in the enhancers. Motif 1 (shown in red characters) is shared among all three retroposon-derived sequences, whereas motif 2 (blue characters) is detected in the SINE sub-element of AS071 as well as nPE1. Motif 3 (green characters) resides in CNE1b but not in the SINE sub-element. (TIF) [file pone.0043785.s005.tif]

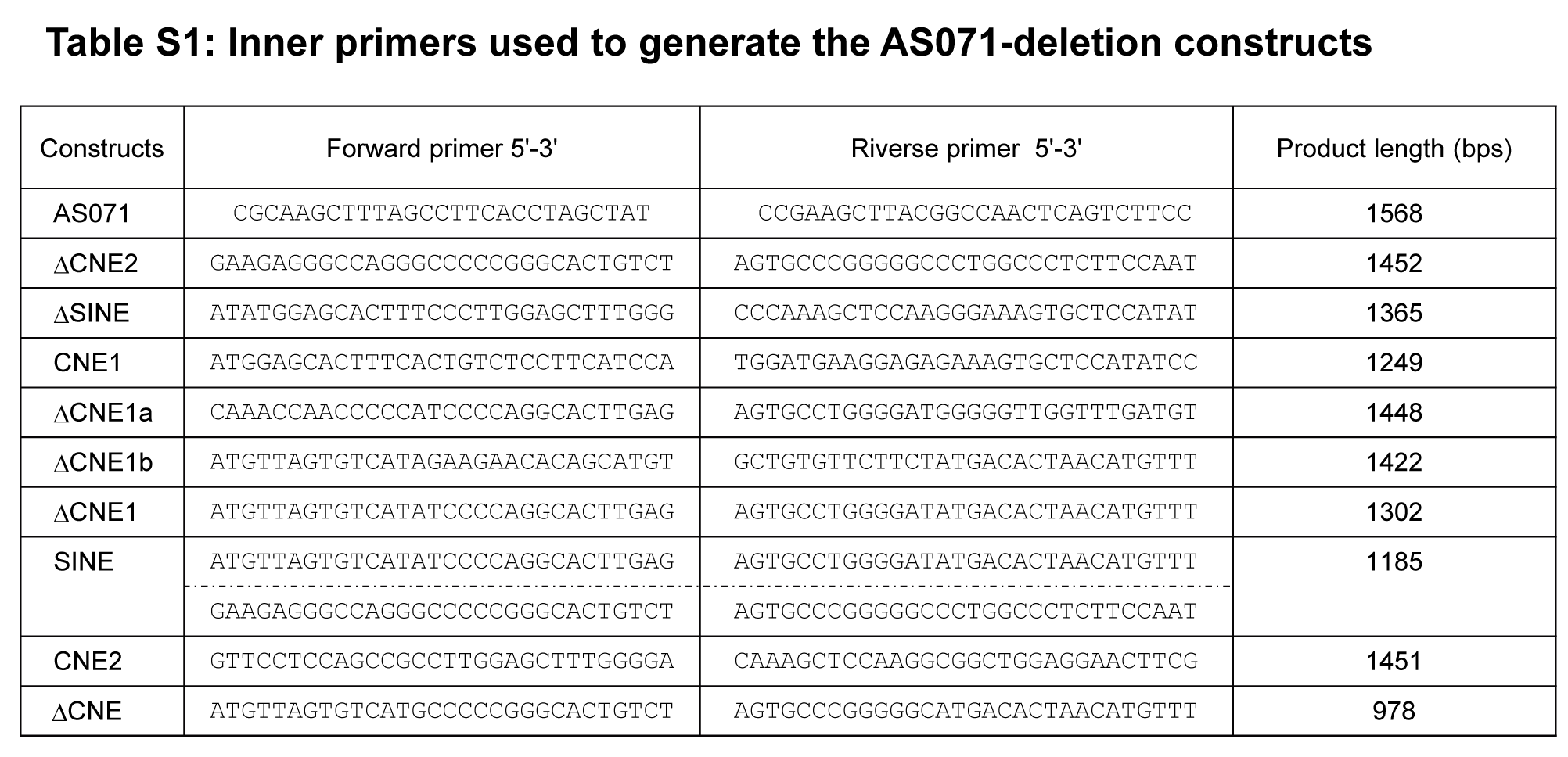

Supplement: Table S1 — Inner primers used to generate the AS071-deletion constructs. A series of AS071-deletion constructs was generated by overlap extension PCR using combinations of inner primers and vector (outer) primers. The length of each final insert fragment is shown in the right column. (TIF) [file pone.0043785.s006.tif]

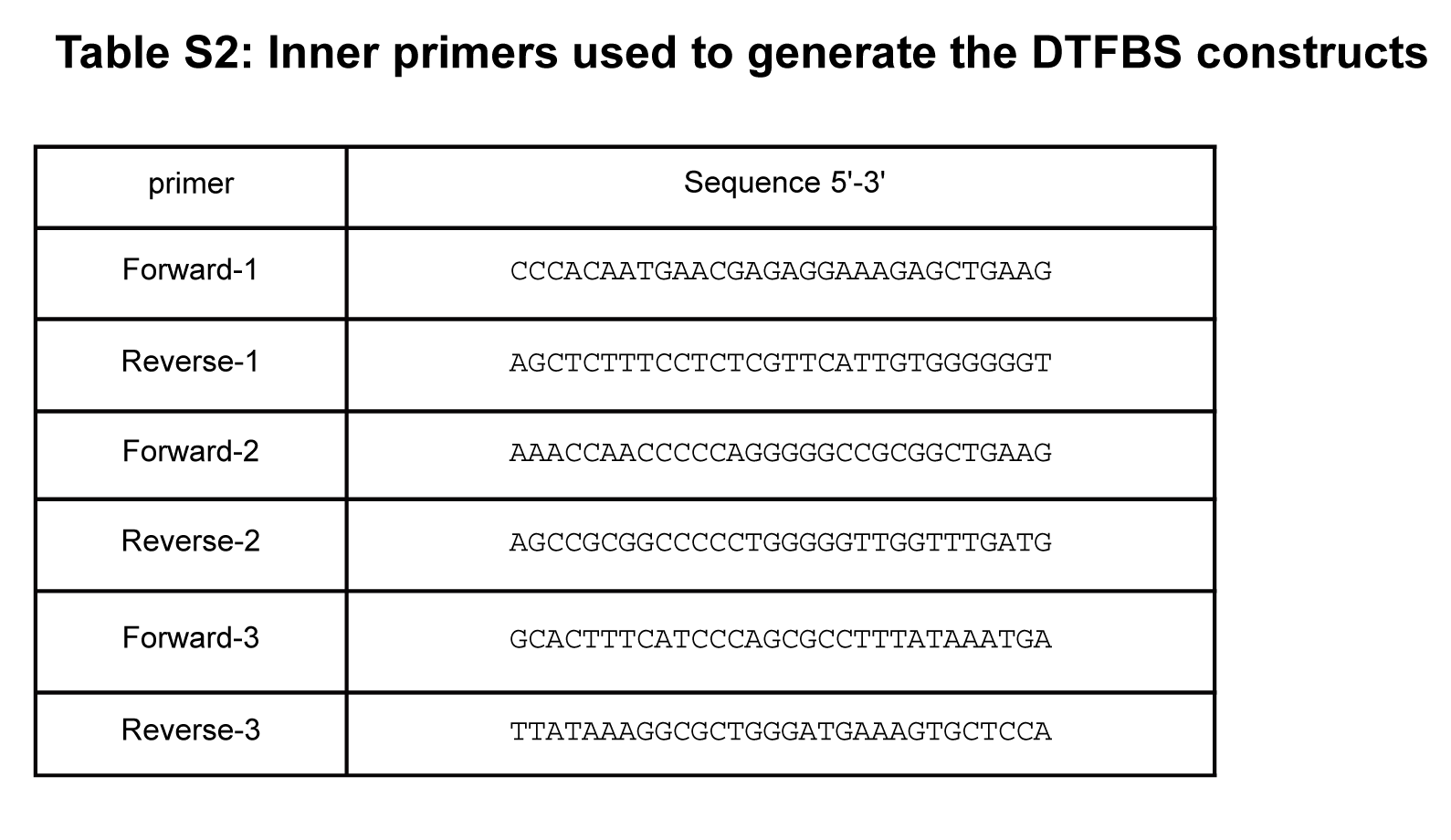

Supplement: Table S2 — Inner primers used to generate the Δ-TFBS constructs. Δ-TFBS constructs were generated by three-step overlap extension PCR using combinations of inner primers and vector (outer) primers. (TIF) [file pone.0043785.s007.tif]
